# Robotic-assisted lung decortication in the management of pleural space complications after lung transplant

**DOI:** 10.1016/j.jhlto.2026.100604

**Published:** 2026-06-08

**Authors:** B.J. Bromberger, Y.B. Gesthalter, A.V. Estrada, N.A. Kolaitis, R.J. Shah, J.P. Singer, L.E. Leard, L. Seijo, J.A. Maheshwari, M.E. Kleinhenz, A. Perez, S.R. Hays, J. Kukreja, B.N. Trinh

**Affiliations:** a Division of Cardiothoracic Surgery, Department of Surgery, University of California, San Francisco, California; b Division of Pulmonary, Critical Care, Allergy, and Sleep Medicine, Department of Medicine, University of California, San Francisco, California; c Division of Thoracic Surgery, Department of Surgery, City of Hope, Irvine, California

**Keywords:** lung transplantation, lung entrapment syndrome, robotic-assisted lung decortication

## Abstract

Robotic-assisted decortication has not been reported in post-lung transplant patients. We reviewed 26 consecutive robotic decortication for lung entrapment syndrome from 2019 to 2024. The time from transplant to decortication was 516 days (IQR 260-887). No thoracotomy conversion was necessary. Median chest tube duration was 4 days (IQR 3-6.5), and median length of stay was 6 days (IQR 3-7). Eighty-eight percent of cases achieved complete lung expansion with a median 315 ml improvement in FVC and 235 ml improvement in FEV1. The most severe Clavien–Dindo complications included 1 take-back for airleak and 1 re-intubation. Robotic-assisted decortication was well tolerated, safe, and effective at removing the fibrotic pleural disease and reversing previously defunctionalized lung.

As many as 45% of post-lung transplant patients may experience a pleural space complication, which frequently necessitates further invasive procedures.^1^ Empyemas, hemothoraces, and chronic or recurrent effusions are detrimental effect to allograft function and survival by restricting lung expansion and leading to lung entrapment syndrome. 16% of patients after lung transplant require decortication, but this procedure is associated with risks, including bleeding, prolonged airleaks, post-operative respiratory failure, and even death.^2^, ^3^ Decortication can be performed through a thoracotomy, but reports on video-assisted thoracoscopic surgery (VATS) showed decreased post-operative pain, reduced length of stay, and reduction in complication rates when a minimally invasive approach was used.^4^, ^5^ Incomplete decortication was reported in 38% with 21% of the cases requiring concurrent pleurodesis and pleurX placement.^3^ Outside of case reports,6., 7 little exists in the literature describing the use of robotic decortication for the management of complex pleural space problems in thoracic patients, and there is an absence of reporting on this approach in post-lung transplantation. The benefits include increased instrument range of motion, surgical visibility, and autotomy.

## Methods

A retrospective study was conducted at a single center (University of California, San Francisco, USA) with lung transplants performed from 2011 to 2024. Patients were transplanted using the bilateral transverse thoracosternotomy (clamshell) approach with basiliximab induction followed by maintenance immunosuppression with prednisone, mycophenolate mofetil, and tacrolimus. Cases of recurrent effusions not responding to tube thoracostomy and/or fibrinolytic therapy were referred for decortication for lung entrapment syndrome. Both forced vital capacity (FVC) and forced expiratory volume in 1 s (FEV1) data were extracted from spirometry testing during routine surveillance. The change of flows (∆FVC or ∆FEV1) was calculated by taking the difference between the nadir just prior to the decortication and about 1 year after decortication. Surgical decortication was performed robotically using a total multi-portal approach with the da Vinci Xi system (Intuitive Surgical, Sunnyvale, California, USA) in the lateral decubitus position with single lung ventilation. The first port was created with a direct cut down into the pleural space followed by bedside thoracoscopic dissection to create space for addition ports, then pressurized to 8 to 10 mm Hg with CO_2_. Adhesions were divided with cautery and lung reduced to allow for hilar mobilization. Surgical planes were identified for decortication which involved the visceral side of the disease. Specimen were collected for microbiology and pathology. All patients were extubated in the operating room. Two chest tubes (1 anterior and 1 over the diaphragm) were managed with a digital device until output was generally less than 200 ml per day and air leak averaged < 20 ml/min (Centese Thoraguard). Pain was managed with local 0.25% bupivacaine, tylenol, oral/intravenous narcotics without nonsteroidal anti-inflammatory drugs. Six of the initial 10 cases had an epidural catheter placed, and the subsequent 16 patients did not require epidurals. Pain management was at the discretion of the medical team.

## Results

**Figure 1:**
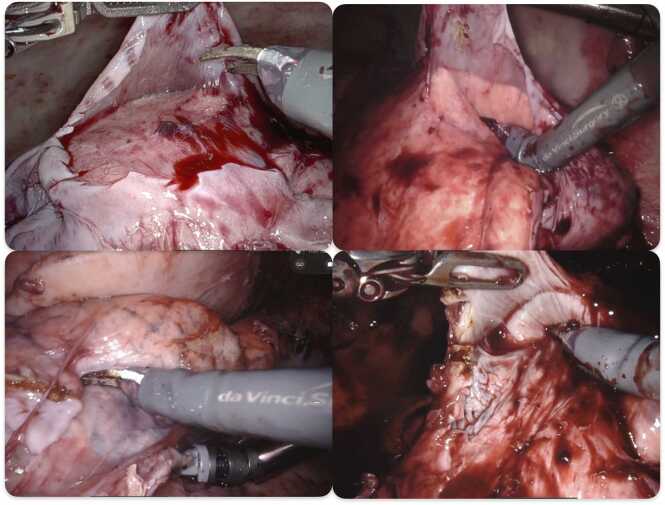
Robotic-assisted decortication technique shows photos of post-transplant cases demonstrating removal of thick pleural disease. An 8 mm monopolar curved scissors and EndoWrist suction irrigator (Intuitive Surgical) were used to take down adhesions and reduce the lung to allow for hilar mobilization. Continuous positive airway pressure (CPAP) with FiO_2_ mixed to room air was used to identify surgical planes

We analyzed 26 consecutive bilateral lung transplant recipients who underwent robotic lung decortication between 2019 and 2024 by one surgeon (BNT). Demographic and clinical data are provided in Table 1. The median age at decortication was 67 years (IQR 63-70) and the majority of patients were male (85%). The median time from transplant to decortication was 516 days (IQR 260-887). Intraoperative findings were often notable for entrapped lungs associated with thick, fibrotic pleural tissue enveloping most commonly the lower lobes and diaphragm (Figure 1).

**Table 1:** Patient Characteristics

	**n**	**%**			**Median**	**IQR**	**Range**
Male	22	85		Age	67	63-70	49-75
Female	4	15		Weight (kg)	88.8	77.1-93.7	59.9-101.7
Indication for transplant				BMI	28.6	25.5-30.1	19.8-36.4
ILD	24	92		Time to decortication (days)	516	260-887	108-3595
COPD	1	4		Albumin (g/dL)	3.8	3.3-4.0	2.2-4.3
Re-do transplant	1	4		Hemoglobin (g/dL)	11.4	10.2-13.5	8.5-15.3
Indication for decortication				Creatinine (mg/dL)	1.32	1.15-1.66	0.49-2.41
Lung entrapment	19	73					
Suspect infection	5	19					
Hemothorax	2	8					
Laterality							
Right	11	42					
Left	14	54					
Both	1	4

Table 2 shows the pathology and microbiology characteristics found at the time of surgery. Twenty cases (77%) had no evidence of acute cellular rejection at the time of decortication, and 54% (14/26) had no history of acute cellular rejection. Only one (1/26) had de novo A2B0 on surgical lung biopsy at the time of decortication. Pleural infection was identified in 23% of the cases (2 fungal and 4 bacterial).

**Table 2:** Pathology and Microbiology Characteristics

**Highest ISHLT Grading Prior to Decortication**	**ISHLT Grading at Time of Decortication**	**n Total = 26**
A0B0	A0B0	14
A0B1R	A0B0	1
A1/2B0	A0B0	5
A1B0	A0B1R	1
A1/2B0	A1B0	3
A2B0	A2B0	1
A0B0	A2B0	1
**Culture negative**		20
**Bacterial**^**#**^		4
**Fungal**^**%**^		2

#Propionibacterium acnes, Staphylococcus epidermidis

%*Candida albicans*, Scopulariopsis spp

Table 3 shows the post-operative characteristics and adverse outcomes. The median estimated blood loss was 100 ml (IQR 50-150). There were no significant intraoperative complications and no conversion to open thoracotomy. The majority of patients went to the floor post-operatively (86%) and the remainder to the ICU (14%). ICU admissions were largely due to initial surgeon’s preference for monitoring and not for end-organ support or hemodynamic instability. The median duration of chest tube was 4 days (IQR 3-6.5) and median length of stay was 6 days (IQR 3-7). Low grade I-II Clavien–Dindo complications included acute kidney injury (2/26), transfusion for acute blood loss anemia (2/26), and air leaks (9/26) as defined by chest tube duration for >5 days. However, 6 of these (6/9) occurred during the first ten cases of the series. Three patients were discharged with a chest tube connected to a Heimlich valve. One moderate grade III Clavien–Dindo complication was identified in a patient requiring take-back surgery to repair a bronchopleural fistula. We had one grade IV complications (re-intubation) and no grade V complication (zero 30-day mortality). One patient died 6 months after decortication secondary to complications from disseminated adenovirus and pneumonia. There were no hospital readmissions within 30 days of decortication and no post-procedure empyema.

**Table 3:** Post Operative Characteristics

	**Mean**	**Median**	**IQR**	**Range**
**EBL (ml)**	129	100	50-150	50-300
**Chest tube (days)**	4.5	4	3-6.5	3-15
**ICU (days)**	0.27	0	0-0	0-3
**LOS (days)**	5.88	6	3-7	3-12
**Adverse outcomes**	**n**	**%**	**CD grade**	
**Open Conversion**	0	0		
**Transfusion**	2	8	II	
**Re-intubation**	1	4	IV	
**Syncopal event**	1	4		
**ATN**	2	8		
**PAL >5 days**	9	35		
**Readmission**	0	0		
**Incomplete lung apposition**	3	12		
**Re-intervention**	1	4	IIIb	
**30 day mortality**	0	0		
**1-year mortality**	1	4		

ATN, acute tubular necrosis; CD, Clavien–Dindo complication grade; EBL, Estimated blood loss; LOS, length of stay; PAL, prolonged air leak

**Figure 2:**
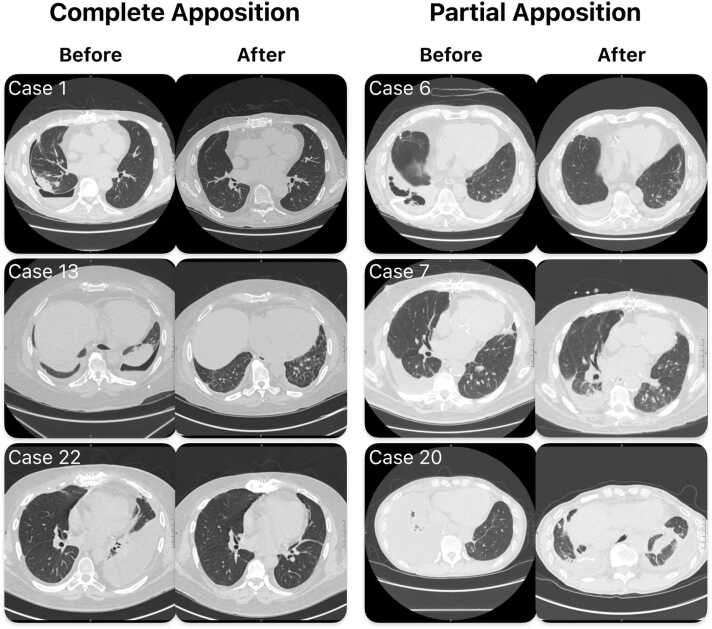
Lung apposition before and after robotic decortication. CT axial views in lung window showing representative cases showing before and 1 year after robotic lung decortication

One year following decortication, we observed consistent improvements in pulmonary function (Table 4) and resolution of pleural space disease. 88% of cases achieved complete pleural apposition (Figure 2). Spirometry testing showed a median 15% improvement of FEV1 (235 ml, IQR 120-440) and 17% improvement of FVC (315 ml, IQR 180-530).

**Table 4:** Spirometric Improvements After Robotic Decortication

	**Median**	**IQR**	**Range**
**∆FVC ml (%)**	315 (16.9)	180-530 (7.5-32.5)	−1250 to 1360 (−45 to 73)
**∆FEV1 ml (%)**	235 (15.2)	120-440 (7-30)	−1090-820 (−42 to 75)

FVC, Forced vital capacity; FEV1, forced expiratory volume in 1 s

## Discussion

We describe, what is to our knowledge, the first case series of a minimally invasive technique using a robot-assisted platform for pleural decortication in post-lung transplant patients. The desire outcomes of a surgical decortication are infection control, pleural space apposition, and improved pulmonary function while minimizing surgical morbidity. While this study did not directly compare robotic to VATS and thoracotomy, we believe that the robotic approach has added values that can be attributed to improved vision, exposure, instrumentation and dexterity. In our experience, moderately severe Clavien–Dindo grade ≥ III complications were limited to under 10%. While prolonged airleak was noted in 9 cases, only 3 patients had prolonged airleaks in the last 16 cases (18%). This is consistent with an initial technology learning curve. Interestingly, these decortications were performed an average of 516 days (IQR 260-887) following the index transplant surgery. We observed that pleural disease presenting this far out is chronic and more fibrotic, and thus more challenging to remove without creating airleaks.^8^ These cases showed a phenotype akin to “chronic organizing empyema” and often the treatment is an “empyemectomy” when feasible. Certainly, aggressive decortication and surgical technique can contribute to airleak severity, and should be balanced with the desire to achieve lung apposition to maximize lung function recovery. Complete lung apposition was achieved in the majority of the cases with measurable spirometric recovery at 1 year. Late decline in spirometric values should be distinguished from FEV1 reduction caused by chronic lung allograft dysfunction (CLAD) as defined by Verleden *et al.*^9^ An understanding of FEV1 class trajectories may have important surveillance implications during the post-transplant period and help to formulate interventions.^10^

## Conclusion

Our findings showed that late occurrence of lung entrapment after lung transplantation can be successfully treated in the majority of the cases while minimizing severe complications. This study has limitations inherent to its retrospective nature. Due to the limited sample size, a direct comparison to VATS and open history cohort was not available. Robotic technology adds to our surgical “tool belt” and we expect it to complement VATS and open approaches well.

## Declaration of competing interest

The authors declare that they have no known competing financial interests or personal relationships that could have appeared to influence the work reported in this paper.
